# Supplementary material for: Functional diversity of brain networks supports consciousness and verbal intelligence
Source: Sci Rep. 2018 Sep 5;8:13259. doi: 10.1038/s41598-018-31525-z (PMC6125486; doi:10.1038/s41598-018-31525-z)
Supplement: Supplementary file 1 — Supporting Information [file 41598_2018_31525_MOESM1_ESM.doc]

**Supporting Information (SI)**

**Title:** Functional diversity of brain networks supports consciousness and verbal intelligence.

**Authors**: Lorina Naci^a*^, Amelie Haugg^b^, Alex MacDonald^c^, Mimma Anello^d^, Evan Houldin^e^, Shakib Naqshbandi^f^, Laura E. Gonzalez-Lara^e^, Miguel Arango^f^, Christopher Harle^f^, Rhodri Cusack^a^, Adrian M. Owen^e^

^a^ Trinity College Institute of Neuroscience, Trinity College Dublin, Dublin, Ireland.

^b^Department of Psychiatry, Psychotherapy and Psychosomatics, Psychiatric Hospital, University of Zurich, Zurich, Switzerland.

^c^Faculty of Medicine, University of Toronto, Toronto, Canada.

^d^Schulich School of Medicine & Dentistry, Western University, London, Canada.

^e^Brain and Mind Institute, Western University, London, Canada.

^f^Department of Anesthesia and Preoperative Medicine, Schulich School of Medicine & Dentistry, Western University, London, Canada.

^*^**Corresponding author:**

Lorina Naci

School of Psychology

Trinity College Institute of Neuroscience

Global Brain Health Institute

Trinity College Dublin

Dublin, Ireland

Telephone: +353 (0)87 688 5642

Email: [nacil@tcd.ie](mailto:nacil@tcd.ie)

**SI Methods**

**Study 1**

**Behavioral Responsivity Tasks inside the MRI Scanner**

*Auditory target detection task.* Participants were instructed via headphones to press a button with their index finger as soon at they heard an auditory beep and to keep their eyes on the fixation cross on the screen. The response box was tapped to the hand to ensure the participant did not loose contact during deep anesthesia (Figure S2). *Memory recall task*. The brief recall test from the Mini Mental State Exam (1) was adopted to test memory function under sedation. The researcher named three unrelated objects clearly and slowly and asked the volunteer to name each of them. The volunteer was instructed to remember the words in order to be able to repeat them in a short while. After 10 minutes, the volunteer was asked the repeat the words. Two different lists (a. Ball- Flag-Tree; b. Flower-Egg- Rope) were used to avoid familiarity effects between the awake and deep anesthesia states. Their presentation was counterbalanced across participants. In the wakeful state all participants remembered all three words (score = 3), and in the deeply anesthetized state, no participant responded to either instructions to name or repeat the words (score =0).

*Resting state protocol.* The eyes-closed resting state condition was consistent with the well-established and most commonly used resting state protocol, including for the purpose of building up massive dataset across different centers around the world (e.g., the 1000 Functional Connectomes Project, http://fcon_1000.projects.nitrc.org) (2), or the BRAIN initiative (Brain Activity Map Project) (3–4). A recent study has pointed to the possibly confounding effect of drifts into sleep that can occur when participants lie at rest with their eyes closed inside the scanner (5). We note that, if the effect of intermittent sleep during the resting state was present in our study, as an effect endemic to the majority of resting state studies, it would therefore not selectively impact on the comparisons we draw with previous literature.

**Supplementary Data**

*Scrambled story condition.* In order to investigate the contributions of the high-level features of the story, including its narrative, to the brain connectivity patterns a novel re-analysis (whole-brain functional connectivity) of the scrambled story condition data collected by Naci et al. (2017) (6) was performed for this study. In order to create the scrambled audio story, the frequencies in the intact audio story were spectrally rotated so the spectro-temporal characteristics of natural speech were maintained but the words were unintelligible, therefore removing the narrative.

**Details of Propofol Administration**

Propofol is commonly used by i.v. infusion for sedation during surgical procedures and for critical care. Propofol is a GABA_A_ potentiating compound. In high doses, propofol is used for induction and maintenance of anesthesia. In accordance with the Canadian Anesthesia Society guidelines, non-invasive blood pressure (NIBP), heart rate, oxygen saturation (SpO2) and end-tital carbon dioxide (ETCO2) were monitored continuously through the use of a dedicated MR compatible anesthesia monitor, and complete resuscitation equipment was present throughout the testing. Supplemental oxygen was administered via nasal cannulae to ensure adequate levels of oxygen at all times and was titrated to achieve an oxygen saturation above 96%. Participants remained capable of spontaneous cardiovascular function and ventilation in deep anesthesia.

Before entering the fMRI scanner, a 20G i.v. cannula was inserted into a vein on the dorsum of the non-dominant hand of the participants. The propofol infusion system was connected to the cannula prior to the first scanning session. No propofol was administered during the “awake” session. In the deep anesthesia session, propofol infusion commenced with a target effect-site concentration of 0.6 µg/ml and oxygen was titrated to maintain SpO2 above 96%. If Ramsay level was lower than 5, the concentration was slowly increased by increments of 0.3 µg/ml with repeated assessments of responsiveness between increments to obtain a Ramsay score of 5. Once Ramsay 5 level of sedation (7) was achieved, the propofol target concentration was kept stable—as determined the TIVA Trainer (the European Society for Intravenous Aneaesthesia, eurosiva.eu) pharmacokinetic simulation program—for the duration of scanning (13 minutes). Throughout the deep anesthesia scanning session, the participant’s behavioral profile was monitored inside the scanner room by the anesthesia nurse and one of the anesthesiologists and outside from the scanner control room, with an infrared camera that displayed the participant’s face. No movement, fluctuations of sedation, or any other state change was observed during the deep anesthesia scanning for any of the participants included in the study.

**fMRI Data Acquisition and Analyses**

*Acquisition*. Noise cancellation headphones (Sensimetrics, S14; www.sens.com) were used for sound delivery. A volume level deemed comfortable by each individual was used for the duration of the experiment. Functional echo-planar images were acquired (33 slices, voxel size: 3 x 3 x 3, inter-slice gap of 25%, TR=2000ms, TE=30ms, matrix size=64x64, FA=75 degrees). The audio story and resting state had 155 and 256 scans, respectively. An anatomical volume was obtained using a T1-weighted 3D MPRAGE sequence (32 channel coil, voxel size: 1 x 1 x 1 mm, TA=5 minutes and 38 seconds, TE=4.25ms, matrix size=240x256x192, FA=9 degrees). *Preprocessing*. The processing steps were: correction for timing of slice acquisition, motion correction, normalization to a template brain, and smoothing. The data were smoothed with a Gaussian smoothing kernel of 10mm FWHM (8). Spatial normalization was performed using SPM8’s segment-and-normalize procedure, whereby the T1 structural was segmented into grey and white matter and normalized to a segmented MNI-152 template. These normalization parameters were then applied to all EPIs. The time series in each voxel was high pass-filtered with a cutoff of 1/128 Hz to remove low-frequency noise, and scaled to a grand mean of 100 across voxels and scans in each session.

*Functional connectivity (FC) analysis*. No global signal regression was performed to avoid the formation of artificial anti-correlations, a confounding effect previously reported (9–10). The following networks, which have consistently been reported in seed-based analyses of resting state studies (11), were investigated: the default mode (DMN), the dorsal attention (DAN), the executive control (ECN), the salience (SAL), the somato-sensory (SM), the visual (VIS) and the auditory (AUD) network (Table S1). To account for the non-normal distribution of correlation values (12), all statistical analyses were performed on z-transformed correlation values, using Fisher’s r-to-z transformation. For visualization purposes, these z-values were eventually re-transformed in correlation values. Within-subjects ANOVAs and paired-samples T-tests were used to explore effects of interest, with bonferroni correction for multiple comparisons.

In the interest of brevity, analyses reported in the main text focused on five networks that are key for processing an auditory narrative (DMN, DAN, ECN, VIS, AUD). A full analysis with the 7 networks showed similar results (Figure S4).

**Study 2**

**Patients** **Behavioral Testing**

The patient’s structural scans are shown in Figure S1, and demographic and clinical data are summarized in Table S2, S3. The CRS-R was not appropriate for administration in the LIS patient, who established communication via eye movements, thus confirming his conscious awareness (noted as ‘n.a.’ in Tables S2, S3).

**Command-following fMRI Paradigm**

*Stimuli*. The stimuli were eleven single words (‘one’, ‘two’, ‘three’, ‘four’, ‘five’, ‘six’, ‘seven’, ‘eight’, ‘nine’, ‘yes’, ‘no’). *Design*. The fMRI selective auditory attention paradigm has been previously described in healthy individuals (13) and patients with DoC (14), and is designed to identify the ability to follow commands to selectively attending to stimuli, by recruiting top-down attention. On each trial, participants were instructed to either count a target word (‘yes’ or ‘no’) presented among pseudorandom distractors (spoken digits one to nine), or to relax. Each trial had an on/off design: sound (~22.5s) followed by silence (10s). The scan lasted five minutes, including instructions. (Figure S5).

**Study 3**

**Participants**

The 16 final participants of the anesthesia study were invited to perform a cognitive battery of tests several weeks later. 2 (female) participants had moved away and could not be enrolled in the experiment.

**Design of Cognitive Testing Battery**

Hampshire et al. (2012) (15) reported the behavioral performance of a large group of people (N=44,600) in the same cognitive battery comprising 12 tasks. Principal component analysis (PCA) of the behavioral data revealed three significant behavioral components that each accounted for more variance than was contributed by any one test (Table S4), and that together accounted for 45% of the total variance (15). The *short-term memory (STM)* component accounted for the variance of all of the tasks in which information was held actively on line in short-term memory. The *reasoning* component accounted for the variance of all of the tasks in which information was transformed in mind according to logical rules. The *verbal* component accounted for the variance in the three tasks that used verbal stimuli (Table S4), these being digit span, verbal reasoning, and color-word remapping. Multiple comparisons in analysis of correlation between cognitive performance and functional connectivity were corrected with the Bonferroni correction.

All tests are available for evaluation at [www.CambridgeBrainSciences.com](http://www.CambridgeBrainSciences.com/). A brief description the each tasks, in alphabetical order, follows.

*Colour-Word Remapping Task.* A variant on the Stroop test (16). Three colored words are displayed on the screen: one at the top and two at the bottom. Participants must indicate which of two colored words at the bottom of the screen correctly describes the color that the word at the top of the screen is written in. The color word mappings may be congruent, incongruent, or doubly incongruent, depending on whether or not the color of the top word matches the color that it is written in. Participants have 90 seconds to solve as many problems as possible.

*Deductive Reasoning Task.* Based on a sub-set of problems from the Cattell Culture Fair Intelligence Test (17). Nine patterns will appear on the screen. The features that make up the patterns are color, shape, and number and are related to each other according to a set of rules. Participants must deduce the rules that relate the object features and select the pattern that do not correspond to those rules. Difficulty is increased or decreased depending on whether the participant got the previous trial correct. Participants have 3 minutes to solve as many problems as possible.

*Digit Span Task.* A variant on the verbal working memory component of the WAIS-R intelligent test (18). A sequence of numbers will appear on the screen one after another. Once the sequence is complete, participants must repeat the sequence by entering them on the keyboard. Difficulty is increased or decreased by one number depending on whether the participant got the previous trial correct. After three errors, the task will end.

*Feature Match Task.* Based on the classical feature search tasks that have been used to measure attentional processing (19). Two grids are displayed on the screen, each containing an array of abstract shapes. In half of the trials the grids differ by just one shape. Participants must indicate whether or not the grid’s contents are identical. Difficulty is increased or decreased by one shape depending on whether the participant got the previous trial correct. Participants have 90 seconds to solve as many problems as possible.

*Interlocking Polygons Task.* Based on the Interlocking Pentagons Task, which is often used in the assessment of age related disorders. A pair of overlapping polygons is displayed on one side of the screen. Participants must indicate whether a polygon displayed on the other side of the screen is identical to one of the interlocking polygons. Difficulty is increased by making the differences between the polygons more subtle or decreased by making the differences between the polygons more pronounced. Participants have 90 seconds to solve as many problems as possible.

*Paired Associates Task.* A variant on a paradigm that is commonly used to assess memory impairments in aging clinical populations (20). Boxes are displayed at random locations on the screen. The boxes are opened one after another to reveal an enclosed object. Subsequently, the objects are displayed in random order in the centre of the screen and participants must determine which box contains the object that is presented. Difficulty is increased or decreased by one box depending on whether the participant got the previous trial correct. After three errors, the task will end.

*Self-Ordered Search Task.* Based on a test that is used to measure strategy during search behaviours (21). Sets of boxes are displayed on the screen in random locations. Participants must find a hidden “token” by clicking on the boxes one at a time to reveal their contents. When the token is found, it is hidden within another box. On any given trial, the token will not appear within the same box twice, thus, participants must search the boxes until the token has been found once within each box. If they search the same empty box twice whilst looking for the token, or search a box in which the token has previously been found, this is an error and the trial ends. Difficulty is increased or decreased by one box depending on whether the participant got the previous trial correct. After three errors, the task will end.

*Spatial Planning Task.* A variant on the Tower of London Task (22), which is used to measure executive function. Numbered beads are positioned on a tree shaped frame. Participants must reposition the beads so that they are configured in ascending numerical order running from left to right and top to bottom of the tree, in as few moves as possible. Problems become progressively harder with the total number of moves required and the planning complexity increasing in steps. Trials are aborted if the participant makes more than twice the number of moves required to solve the problem. Participants have 3 minutes to solve as many problems as possible.

*Spatial Rotation Task.* Often used for measuring the ability to manipulate objects spatially in mind (23). Two grids of colored squared are displayed to either side of the screen with one of the grids rotated by a multiple of 90 degrees. When rotated, the grids are either identical or differ by the position of just one square. Participants must indicate whether or not the grids are identical. Participants have 90 seconds to solve as many problems as possible.

*Spatial Span Task.* A variant on the Corsi Block Tapping Task (24), used for measuring spatial short-term memory capacity. 16 squares are displayed in a 4 x 4 grid. A sub-set of the squares will flash in a random sequence at a rate of 1 flash every 900 ms. Subsequently, participants must repeat the sequence by clicking on the squares in the same order in which they flashed. Difficulty is increased or decreased by one box depending on whether the participant got the previous trial correct. After three errors, the task will end.

*Verbal Reasoning Task.* Based on Alan Baddeley’s three minute grammatical reasoning test (25). Short sentences describing the relationship of two shapes along with an image of the shapes are displayed on the screen. Participants must indicate whether the sentence correctly describes the pair of objects displayed on the screen. Participants have 90 seconds to solve as many problems as possible.

*Visuo-spatial Working Memory Task.* A variant on a task from the non-human primate literature (26). Sets of numbered squares are displayed on the screen at random locations. After a variable interval of time, the numbers disappear leaving just the blank squares and participants must respond by clicking the squares in ascending numerical sequence. Difficulty is increased or decreased by one numbered box depending on whether the participant got the previous trial correct. After three errors, the task will end.

**SI Results**

**Study 1**

**Network connectivity in the wakeful state during the audio story and resting state**

During the audio story in the wakeful state, brain connectivity showed a distinctive structure of network pairings and dissociations. The fronto-parietal networks (dorsal attention [DAN] and executive control [ECN]) were most strongly connected to one another than all other networks [t(15)=5.86, p<0.0001; t(15)=5.64, p<0.0001, respectively] and weakly connected to the default mode network (DMN) (Figure S3A, C). This network organization was expressed over and above the inherent connectivity profile observed in the resting state (Figure S2A–C), suggesting that it was driven by the processing of sensory and higher-order features of the story. Critically, this structure was lost in deep sedation (Figure 3A, B).

**Effect of anesthesia during the audio story condition on *within* network connectivity**

We found a main effect of network [F(12)= 11, p=0.001] and a state x network interaction effect [F(12)= 3.72, p=0.034]. Two tailed T tests showed that the connectivity *within* the nodes of VIS increased significantly [t(15)= 2.7, p<0.05] in deep anesthesia relative to wakefulness. The DAN showed a similar but weaker (tend) effect [t(15)= 1.9, p=0.07]. The connectivity *within* the other three networks remained unchanged.

**Effect of anesthesia during the audio story condition on *between* network connectivity**

We found a main effect of state [F(15)=14.67, p=0.002], network [F(12)= 65.64, p<0.0001], and a state x network interaction effect [F(12)= 6.34, p=0.006]. Two tailed T tests showed that connectivity *between* each individual network and the rest of the other networks increased significantly in deep anesthesia relative to wakefulness: DMN [t(15)= 4.5, p<0.0001], DAN [t(15)= 2.8, p<0.05], ECN [t(15)= -3.92, p=0.001], VIS [t(15)= 2.59, p<0.05] and AUD [t(15)= 4.06, p=0.001].

**Effect of anesthesia during the audio story condition functional differentiation among networks calculates as the ratio: *between-/within-connectivity***

The *between-* connectivity represented the average value of the connectivity of one network to all other networks, whereas the *within-* connectivity represented the strength of connectivity within the nodes of one network to one another. In this measure, a higher *between-/within*-connectivity value represents lower differentiation of one network from the other networks. A 2x2 repeated measures ANOVA with factors state (awake, deep) and networks (DMN, DAN, ECN, VIS, AUD) revealed a main effect of state [F(15)=11.4; p<0.005], network [F(15)=7.2; p<0.05], and state x network interaction effect [F(15)=36.2; p<0.001]. The interaction effect was driven by the connectivity ratio increasing (or, the functional differentiating decreasing) in the deep anesthesia state compared to the wakeful state.

**Effect of anesthesia during the resting state condition on *within* network connectivity**

We found a main effect of network [F(12)=12.6, p<0.0001] and a weak (trend) effect of *state,* driven by the decrease of connectivity *within* the nodes of each network to one another [F(15)=3.63, p=0.08]. Two tailed T tests showed that the connectivity *within* the nodes of the DMN increased significantly in deep anesthesia state relative to wakefulness [t(15)=2.3, p<0.05]. Similarly, all other networks apart for the visual showed decreased *within* network connectivity in deep anesthesia, but weaker (trend) effect: DAN: t(15)=1.81, p=0.09; ECN: t(15)=1.77, p=0.097; AUD: t(15)=1.93, p=0.07.

**Effect of anesthesia during the resting state condition on *between* network connectivity**

We found a state x network interaction effect [F(12)=4.83, p<0.05]. This was driven by the differential effect of deep anesthesia on the five networks. Some of the networks showed a trend toward lower connectivity in the deep anesthesia state relative to wakefulness, whereas others stayed the same. None of five networks’ *between* connectivity, or the connectivity of itself with all the other the networks, changed in deep anesthesia relative to wakefulness.

**Effect of anesthesia during the resting state condition functional differentiation among networks calculates as the ratio: *between-/within-connectivity***

A 2x2 repeated measures ANOVA with factors state (awake, deep) and networks (DMN, DAN, ECN, VIS, AUD) revealed a main effect of network [F(15)=; p<], driven by the fact that across both states, some networks had higher *between-/within-* connectivity ratios, or a weaker differentiation value than other networks. Unlike the audio story condition (above), no effect of anesthesia or interactions between networks and state were observed.

**Effect of anesthesia on the connectivity between the thalamus and cortical networks**

To test whether any of the changes observed on the cortico-cortical connectivity related to gating of sensory processes at the thalamic level, we investigate the connectivity between the thalamus and each cortical network. The thalamus ROI was independently defined based on landmark coordinates from the same literature source as the ROIs comprising each reported network (11). The connectivity of the thalamus ROI to the five networks was unaffected by anesthesia, either in the audio story condition or in the resting state (Figure S7).

**Study 2**

**Brain-damage profiles in individual patients**

3/11 patients (P4, P8, P9; all DoC-) showed very limited to absent grey matter mass in auditory and fronto-parietal cortical regions, and overall had extensive atrophy, emaciated cortical mantle and highly enlarged ventricles. The other 8/11 patients had preservation in the auditory and fronto-parietal cortical regions and elsewhere in the brain (Figure S1).

**Auditory processing in severely brain-injured patients**

Prior to assessing command-following, we assessed auditory perception to ensure that it could not have been a limiting factor to producing willful brain responses. Of the patients in the unconscious group (DoC-)—who met the clinical criteria for the diagnosis of unconsciousness provided from their medical doctors and showed no additional evidence of covert awareness in the fMRI-based command-following protocol (Figure 6, last two columns)— 2/5 (P1 and P7) showed evidence of basic auditory processing, but no evidence of higher-order cognition (Figure 6). Therefore, consciousness could not have been ruled out on the basis of impaired sensory processing in these patients. In the remaining 3/5 patients (P4, P8, P9), the aforementioned pervasive brain degeneration (the worst in the group) suggested that each individual’s brain was incapable of supporting auditory processing, as well as any other perceptual and cognitive processes including conscious awareness.

**SI References**

1. Folstein, M.F., Folstein, S.E., and McHugh, P.R. (1975). “Mini-mental state”: a practical method for grading the cognitive state of patients for the clinician. Journal of psychiatric research 12(3), 189-198.
2. Biswal BB et al. (2010)Toward discovery science of human brain function. Proc Natl Acad Sci U S A. 107(10):4734-9.
3. Alivisatos., A.P. et al. (2012). The brain activity map project and the challenge of functional connectomics. Neuron 74(6):970-4.
4. Devor A. et al. (2013). The challenge of connecting the dots in the B.R.A.I.N. Neuron. 80(2):270-4.
5. Tagliazucchi., E, Laufs., H. (2014). Decoding wakefulness levels from typical fMRI resting-state data reveals reliable drifts between wakefulness and sleep. Neuron 82(3):695-708.
6. Naci, L., Sinai, L, Owen, A. M. (2017) Detecting and interpreting conscious experiences in behaviorally non-responsive patients. NeuroImage. 145(Pt B):304-313.
7. Ramsay, M., Savage, T., Simpson, B., and Goodwin, R. (1974). Controlled sedation with alphaxalone-alphadolone. Br. Med. J. 2, 656–659.
8. Peigneux, P., et al. (2006). Offline persistence of memory-related cerebral activity during active wakefulness. PLoS Biol. 4(4), e100.
9. Anderson, J.S., et al. (2011). Network anticorrelations, global regression, and phase-shifted soft tissue correction. Human brain mapping 32(6), 919-934.
10. Murphy, K., Birn, R.M., Handwerker, D.A., Jones, T.B., and Bandettini, P.A. (2009). The impact of global signal regression on resting state correlations: are anti-correlated networks introduced?. Neuroimage 44(3), 893-905.
11. Raichle, M.E. (2011). The restless brain. Brain connectivity 1, 3-12.
12. Fisher, R.A. (1915). Frequency distribution of the values of the correlation coefficient in samples from an indefinitely large population. Biometrika 10(4), 507-521.
13. Naci, L., Cusack, R., Jia, V.Z., and Owen, A.M. (2013). The brain's silent messenger: using selective attention to decode human thought for brain-based communication. The Journal of Neuroscience 33(22), 9385-9393.
14. Naci, L., and Owen, A.M. (2013). Making every word count for nonresponsive patients. JAMA Neurol. 70, 1235–41.
15. Hampshire, A., Highfield, R.R., Parkin, B.L., and Owen, A.M. (2012). Fractionating human intelligence. Neuron 76(6), 1225-1237.
16. Stroop, J.R. (1935). Studies of interference in serial verbal reactions. Journal of experimental psychology 18(6), 643.
17. Cattell, R.B. (1949). Culture Free Intelligence Test, Scale 1, Handbook (Champaign, Illinois: Institute of Personality and Abilit).
18. Weschler, D. (1981). Wechsler Adult Intelligence Scale--Revised (The Psychological Corporation).
19. Treisman, A.M., and Gelade, G. (1980). A feature-integration theory of attention. Cogn Psychol 12, 97-136.
20. Gould, R.L., et al. (2005). Functional neuroanatomy of successful paired associate learning in Alzheimer's disease. Am J Psychiatry 162, 2049-2060.
21. Collins, P., Roberts, A.C., Dias, R., Everitt, B.J., and Robbins, T.W. (1998). Perseveration and strategy in a novel spatial self-ordered sequencing task for nonhuman primates: effects of excitotoxic lesions and dopamine depletions of the prefrontal cortex. Journal of cognitive neuroscience 10, 332-354.
22. Shallice, T. (1982). Specific impairments of planning. Philos Trans R Soc Lond B Biol Sci 298, 199-209.
23. Silverman, I.I., et al. (2000). Evolved mechanisms underlying wayfinding. further studies on the hunter-gatherer theory of spatial sex differences. Evol Hum Behav 21, 201-213.
24. Corsi, P.M. (1972). Human memory and the medial region of the brain. .In PhD thesis (Montreal, McGill).
25. Baddeley, A.D. (1968). A three-minute reasoning test based on grammatical transformation. Psychometric science 10, 341-342.
26. Inoue, S., and Matsuzawa, T. (2007). Working memory of numerals in chimpanzees. Curr Biol 17, R1004-1005.

**SI Tables and Figures**

**Table S1.** Overview of selected regions of interests for seven key functional networks (11). IPS: Intraparietal sulcus, MT: Middle temporal area, SMA: Supplementary motor area, PFC: Prefrontal cortex, V1: Primary visual cortex, A1: Primary auditory cortex

| **Network/Region** | **ROI** | **MNI coordinates** | | |
| --- | --- | --- | --- | --- |
| **Default Mode Network** | Posterior cingulate/precuneus Medial prefrontal Left lateral parietal Right lateral parietal Left inferior temporal Right inferior temporal | 0 -1 -46 49 -61 58 | -52 54  -66  -63  -24  -24 | 27  27  30  33  -9  -9 |
| **Dorsal Attention Network** | Left frontal eye field Right frontal eye field Left posterior IPS Right posterior IPS Left anterior IPS  Right anterior IPS Left MT  Right MT | -29  29  -26  26  -44  41  -50  53 | -9  -9  -66  -66  -39  -39  -66  -63 | 54  54  48  48  45  45  -6  -6 |
| **Executive Control Network** | Dorsal medial PFC Left anterior PFC Right anterior PFC Left superior parietal Right superior parietal | 0  -44  44  -50  50 | 24  45  45  -51  -51 | 46  0  0  45  45 |
| **Salience Network** | Dorsal anterior cingulate  Left anterior PFC  Right anterior PFC  Left insula  Right insula  Left lateral parietal  Right lateral parietal | 0  -35  32  -41  41  -62  62 | 21  45  45  3  3  -45  -45 | 36  30  30  6  6  30  30 |
| **Sensorimotor Network** | Left motor cortex  Right motor cortex  SMA | -39  38  0 | -26  -26  -21 | 51  48  48 |
| **Visual Network** | Left V1 Right V1 | -7  7 | 83  83 | 2  2 |
| **Auditory Network** | Left A1  Right A1 | -62  59 | -30  -27 | 12  15 |
| **Thalamus** |  | 0 | -12 | 9 |

**Table S2. Patients’ demographic, clinical and fMRI assessment data. M: Male, F: Female, VS: Vegetative State, MCS: Minimally Conscious State, LIS: Locked-In Syndrome**

| **Patient no.** | **Gender/**  **Age**  ***ys*** | **Diagnosis** | **Time post injury**  ***mo*** | **CRS-R** | **Aetiology** | **Behavior**  **Movement to command** | **Imaging**  **Command following** |
| --- | --- | --- | --- | --- | --- | --- | --- |
| 1 | M/27 | VS | 89 | 7 | Traumatic brain injury secondary to motor vehicle accident | No | No |
| 2 | F/44 | VS | 248 | 3 | Traumatic brain injury secondary to motor vehicle accident | No | Yes |
| 3 | F/46 | MCS | 234 | 8 | Non-traumatic brain injury from near drowning | No | Yes |
| 4 | F/35 | VS | 25 | 5 | Non-traumatic anoxic brain injury secondary to bilateral pulmonary emboli and cardiac arrest | No | No |
| 5 | M/19 | VS | 3 | 6 | Non-traumatic anoxic brain injury secondary to aspiration pneumonia | No | Yes |
| 6 | F/25 | MCS | 69 | 9 | Traumatic brain injury secondary to motor vehicle accident | No | Yes |
| 7 | F/49 | MCS | 148 | 15 | Traumatic brain injury secondary to motor vehicle accident | Yes | No |
| 8 | M/20 | VS | 49 | 5 | Non-traumatic brain injury secondary to Commodio Cordis | No | No |
| 9 | F/51 | VS | 11 | 4 | Non-traumatic anoxic brain injury secondary to cardiac arrest | No | No |
| 10 | M/40 | MCS | 38 | 7 | Traumatic brain injury secondary to motor vehicle accident | No | Yes |
| 11 | M/55 | LIS | 18 | n.a. | Brainstem infarct related to vertebral artery thrombosis | Yes | Yes |

**Table S3.** Coma Recovery Scale-Revised subscale scores for each patient immediately prior to their fMRI assessment. VS: Vegetative State, MCS: Minimally Conscious State, LIS: Locked-In Syndrome

| **Patient no** | **Diagnosis** | **Auditory** | **Visual** | **Motor** | **Oromotor/**  **verbal** | **Communi-**  **cation** | **Arousal** |
| --- | --- | --- | --- | --- | --- | --- | --- |
| 1 | VS | 1 - Auditory startle | 1 - Visual startle | 2 - Flexion withdrawal | 1 - Oral reflexive | 0 - None | 2 - Eye opening without stimulation |
| 2 | VS | 0 - None | 1 - Visual startle | 0 - None | 0 - None | 0 - None | 2 - Eye opening without stimulation |
| 3 | MCS | 1 - Auditory startle | 3 - Visual pursuit | 2 - Flexion withdrawal | 1 - Oral reflexive | 0 - None | 1 - Eye opening with stimulation |
| 4 | VS | 0 - None | 0 - None | 2 - Flexion withdrawal | 1 - Oral reflexive | 0 - None | 2 - Eye opening without stimulation |
| 5 | VS | 2 - Localization to sound | 1 - Visual startle | 1 - Abnormal posturing | 0 - None | 0 - None | 2 - Eye opening without stimulation |
| 6 | MCS | 2 - Localization to sound | 3 - Visual pursuit | 1 - Abnormal posturing | 1 - Oral reflexive | 0 - None | 2 - Eye opening without stimulation |
| 7 | MCS | 4 - Consistent movement to command | 4 - Object localization: reaching | 4 - Automatic motor response | 1 - Oral reflexive | 1 - Non-functional: intentional | 1 - Eye opening with stimulation |
| 8 | VS | 1 - Auditory startle | 1 - Visual startle | 0 - None | 1 - Oral reflexive | 0 - None | 2 - Eye opening without stimulation |
| 9 | VS | 1 - Auditory startle | 0 - None | 1 - Abnormal posturing | 1 - Oral reflexive | 0 - None | 1 - Eye opening with stimulation |
| 10 | MCS | 1 - Auditory startle | 3 - Visual pursuit | 1 - Abnormal posturing | 1 - Oral reflexive | 0 - None | 1 - Eye opening with stimulation |
| 11 | LIS | n.a. | n.a. | n.a. | n.a. | n.a. | n.a. |

**Table S4**. Task-component loadings from the PCA of a large group (N=44,600) behavioral data with orthogonal rotation. Adapted with permission from Hampshire et al., 2012.

|  |  | **STM** | **Reasoning** | **Verbal** |
| --- | --- | --- | --- | --- |
| Spatial span |  | 0.69 | 0.22 |  |
| Visuospatial working memory |  | 0.69 | 0.21 |  |
| Self-ordered search |  | 0.62 | 0.16 | 0.16 |
| Paired associates |  | 0.58 |  | 0.25 |
| Spatial planning |  | 0.41 | 0.45 |  |
| Spatial rotation |  | 0.14 | 0.66 |  |
| Feature match |  | 0.15 | 0.57 | 0.22 |
| Interlocking polygons |  |  | 0.54 | 0.3 |
| Deductive reasoning |  | 0.19 | 0.52 | –0.14 |
| Digit span |  | 0.26 | –0.2 | 0.71 |
| Verbal reasoning |  |  | 0.33 | 0.66 |
| Color-word remapping |  | 0.22 | 0.35 | 0.51 |

**Figure S1.** **Patient structural images**. The sagittal left and right, coronal and horizontal slices of each patient’s structural scan are displayed. The posterior commissure in each patient’s native space was used as a reference point for the coronal and horizontal slices. The radiologist’s notes on the clinical CT/ MRI scans briefly describe extent and nature of damage. 3/11 patients (P4, P8, P9; all DoC-) showed very limited to absent grey matter mass in auditory and fronto-parietal cortical regions, and overall had extensive atrophy, emaciated cortical mantle and highly enlarged ventricles. The other 8/11 patients had preservation in the auditory and fronto-parietal cortical regions and elsewhere in the brain. VS: Vegetative State; MCS: Minimally Conscious State; LIS: Locked-in Syndrome.


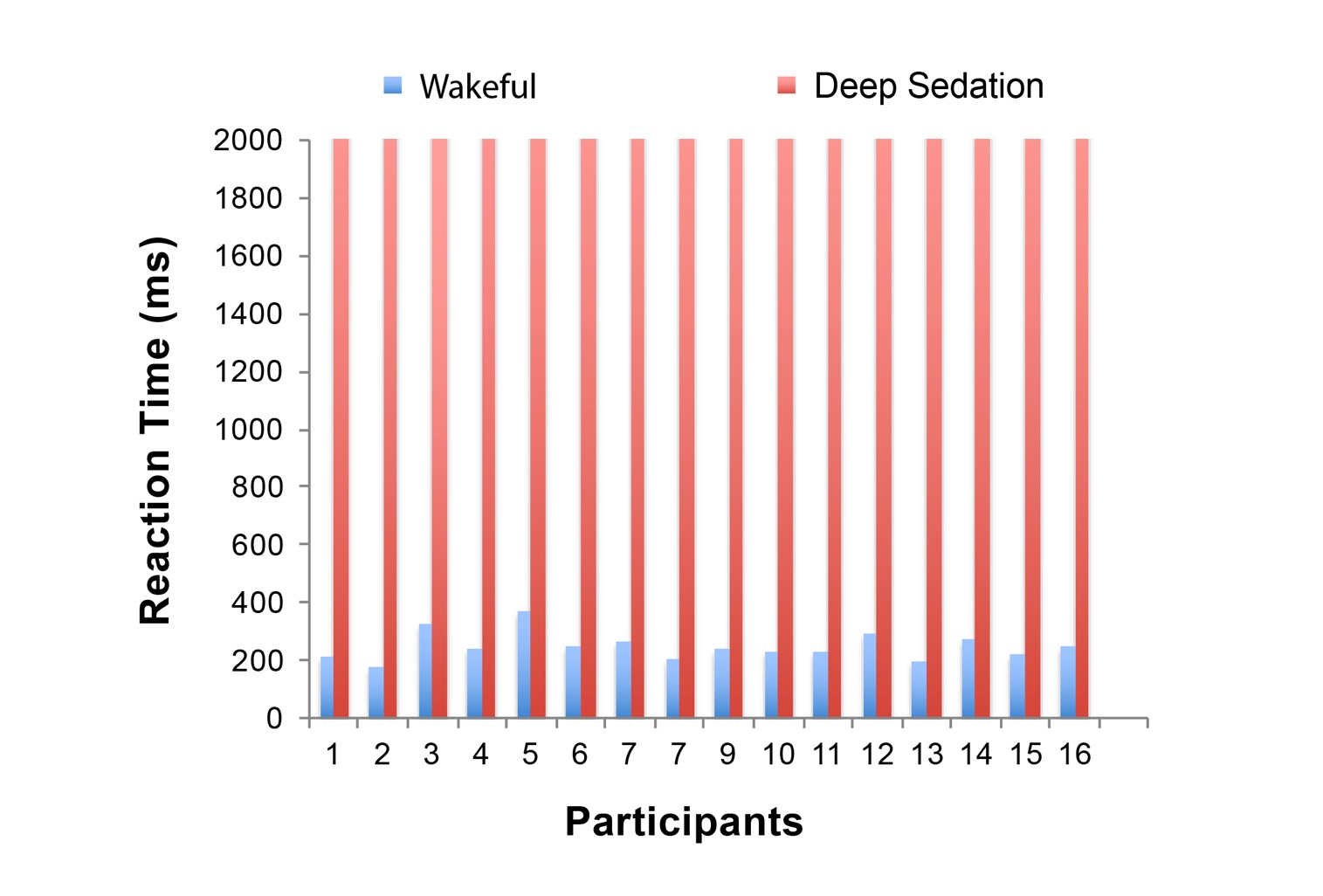


**Figure S2**. **Behavioral response in the target detection task inside the scanner**. Single-subject reaction times (ms) averaged over 50 trials requiring the detection of the auditory target, in the wakeful (blue) and deeply anesthetized states (red). In deep sedation, all participants provided no response within 2000ms.

**Figure S3. Network connectivity in the wakeful state**. (A–B) Correlation matrices for the audio story and resting state conditions. The cells *in*/*off* the middle diagonal of each matrix represent the average connectivity between ROIs within a network/pair-wise connectivity between the ROIs of different networks. Warm/cool colors depict high/low connectivity, as seen in the heat-bar scale DMN/DAN/ECN/VIS/AUD=Default Mode/Dorsal Attention/Executive Control/Visual/Auditory network Bar-graphs depict the connectivity between the DAN and ECN, the DAN and all others, and the ECN and all other networks, for each condition. (C).

**Figure S4. Global *within*- and *between*-network functional connectivity perturbations by propofol for seven networks. (**A–D) Functional connectivity matrices for seven brain networks in the story and resting state conditions, in the wakeful and deep sedation states. Each cell represents the correlation of the time-course of one ROI with another or itself (in the center diagonal). Cells representing correlations of ROIs within each network are delineated by red squares. Warm/cool colors represent high/low correlations, as shown in heat-bar scale. (E) Averaged connectivity (z values) *within-* and *between-*networks in the wakeful (W) and the deep sedation (D) states, during the story and resting state conditions. DMN/DAN/ECN/SAL/SM/VIS/AUD=Default Mode/Dorsal Attention/Executive Control/Salience/Somatosensory/Visual/Auditory network.

**Figure S5**. **Command-following fMRI paradigm.** fMRI paradigm for testing covert awareness in patients with disorders of consciousness. The figure illustrates the design of the two components of the fMRI paradigm: (a) sound perception and (b) command-following. Adapted with permission from: Naci and Owen (2013) (16). Making every word count for nonresponsive patients. JAMA Neurol. 70, 1235–41.

**Figure S6. Patients’ behavioral and neuroimaging response profile**. Neuroimaging response in the Taken story. The patients’ clinical assessment data (columns 2-4) are the same as displayed in Figure 6. The last two columns display lateral images of the patient’s synchronization response to the auditory and fronto-parietal independent components observed in the healthy participants.

**Figure S7. Effect of sedation on thalamo-cortical connectivity.** The left/right panel displays the connectivity of the thalamus ROI and each of the five cortical networks during the audio story/resting state in the awake (blue) and deep sedation condition (orange). We found no effect of sedation the connectivity between the thalamus ROI and each of the five networks during the audio story or the resting state condition.
